# Evaluation of the Probiotic Properties and the Capacity to Form Biofilms of Various *Lactobacillus* Strains

**DOI:** 10.3390/microorganisms8071053

**Published:** 2020-07-15

**Authors:** Célia Chamignon, Virgile Guéneau, Sonia Medina, Julien Deschamps, Angel Gil-Izquierdo, Romain Briandet, Pierre-Yves Mousset, Philippe Langella, Sophie Lafay, Luis G. Bermúdez-Humarán

**Affiliations:** 1 MIcalis Institute, AgroParisTech, INRAE, Université Paris-Saclay, 78350 Jouy-en-Josas, France; celia.chamignon@inrae.fr (C.C.); virgile.gueneau@inrae.fr (V.G.); julien.deschamps@inrae.fr (J.D.); romain.briandet@inrae.fr (R.B.); philippe.langella@inrae.fr (P.L.); 2 INDIGO Therapeutics, 33000 Bordeaux, France; py.mousset@gynov.com (P.-Y.M.); s.lafay@jenwin.fr (S.L.); 3 Research Group on Quality, Safety and Bioactivity of Plant Foods. Department of Food Science and Technology, CEBAS-CSIC, Campus de Espinardo 25, 30100 Murcia, Spain; smescudero@cebas.csic.es (S.M.); angelgil@cebas.csic.es (A.G.-I.)

**Keywords:** *Lactobacillus*, probiotics, permeability, biofilm, stress tolerance

## Abstract

Over the last 20 years, *Lactobacillus* species inhabiting the gastrointestinal tract (GIT) have received much attention, and their health-promoting properties are now well-described. Probiotic effects cannot be generalized, and their uses cover a wide range of applications. It is thus important to proceed to an accurate selection and evaluation of probiotic candidates. We evaluate the probiotic potential of six strains of *Lactobacillus* in different in vitro models representing critical factors of either survival, efficacy, or both. We characterized the strains for their ability to (i) modulate intestinal permeability using transepithelial electrical resistance (TEER), (ii) form biofilms and resist stressful conditions, and (iii) produce beneficial host and/or bacteria metabolites. Our data reveal the specificity of *Lactobacillus* strains to modulate intestinal permeability depending on the cell type. The six isolates were able to form spatially organized biofilms, and we provide evidence that the biofilm form is beneficial in a strongly acidic environment. Finally, we demonstrated the ability of the strains to produce γ-aminobutyric acid (GABA) that is involved in the gut-brain axis and beneficial enzymes that promote the bacterial tolerance to bile salts. Overall, our study highlights the specific properties of *Lactobacillus* strains and their possible applications as biofilms.

## 1. Introduction

For centuries, lactic-acid bacteria (LAB) have been widely used as starters cultures in food fermentation and dairy products. *Lactobacillus* species have been largely studied as part of a healthy gut microbiota, and investigators have proposed their uses as probiotics: live microorganisms that confer a health benefit to the host when administered in appropriate amounts [1]. Although the underlying interactions with the host are strain-dependent, the general mechanisms of action by which probiotics exert their beneficial effects include enhancement of the intestinal epithelial barrier, the ability to adhere to the intestinal mucosa, the competitive exclusion of pathogens, the production of antimicrobial substances, and modulation of the immune system [2].

In their natural habitats, the microorganisms are found in a spatially organized form called biofilms, “a microbially derived sessile community characterized by cells that are irreversibly attached to a substratum or interface or to each other and are embedded in a matrix of extracellular polymeric substances that they have produced” [3]. The biofilm form provides the bacteria with several properties, such as tolerance to the action of antimicrobial peptides and other stressful conditions of the body [4], as well as improving the health of the host in the case of *Lactobacillus spp*. through their beneficial effects. Several studies have shown that the biofilm formation can boost the probiotic activity by enhancing immunomodulatory properties and intestinal permeability, among other actions [5,6].

Dysbiosis of the gut microbiota has been clearly linked to diverse pathological conditions, such as irritable bowel syndrome (IBS) and inflammatory bowel diseases (IBD), for which a direct connection has been established, as well as in less obvious disorders, such as metabolic syndrome or behavioral disorders (depression and autism spectrum disorders) [7]. Several studies, from in vitro experimentation to clinical trials, have demonstrated a key role of intestinal permeability in these diseases. Moreover, a beneficial effect of *Lactobacillus* species on intestinal permeability has been demonstrated in several conditions, such as obesity [8], IBS [9], alcoholic liver disease [10], and stress-mediated alterations and associated visceral sensitivity [11]. The manipulation of intestinal permeability can thus be proposed as a target not only for the prevention but, also, the therapy of human diseases [12].

Due to the wide range of applications of probiotics and their various mechanisms of action, the FAO/WHO has proposed a “Guidelines for Evaluation of Probiotics in Food” [13], suggesting safety and effectiveness criteria for their evaluation. These criteria include a resistance to the stressful conditions encountered in the human body, the ability to adhere to the mucosa, the secretion of antimicrobial molecules, a safety assessment, and evidence of a benefit for human health [14].

We aimed to determine the potential probiotic properties of six different *Lactobacillus* strains. We selected the *Lactobacillus* strains on their ability to enhance intestinal permeability using the transepithelial electrical resistance (TEER) of Caco-2 and T84 intestinal epithelial cells. We then evaluated some characteristics of the probiotic candidates, such as their capacity to adhere on the intestinal epithelial cells Caco-2 and HT-29 MTX and to produce beneficial host/bacteria metabolites. We further characterized the strain by evaluating their capacity to form biofilms and the benefits associated with this microbial lifestyle versus a planktonic lifestyle.

## 2. Materials and Methods

### 2.1. Bacterial Strains and Growth Conditions

The origin, identity, and growth conditions of the strains that were used are presented in Table 1. Strains were stored at −80 °C in PBS X-1 + 16% (*v*/*v*) glycerol. Before experiments, strains were grown overnight, under their respective growth conditions, from frozen stocks and passaged one time before use. In all experiments, the strain of *Lactobacillus rhamnosus* GG was used as a positive control. 

### 2.2. Cell Cultures

The human colorectal adenocarcinoma cell lines Caco-2 and T84 and the mucus-producing cell line HT-29 MTX were used. The Caco-2 cell line was originally obtained from the American Type Tissue Collection (ATCC^®^ HTB-37™), T84 cells were obtained from the European Collection of Authenticated Cell Cultures (ECACC, 88021101), and HT-29 MTX cells were obtained from the Sloan Kettering Memorial Cancer Center (SKMCC). Caco-2 and HT-29 MTX cells were grown in Dulbecco’s modified Eagle’s medium (DMEM), supplemented with 10% heat-inactivated fetal bovine serum, 1% nonessential amino acids (except for HT-29 MTX), and 1% streptomycin/penicillin. T84 cells were maintained in Nutrient Mixture Food F12 (DMEM/F12), supplemented with 6% heat-inactivated fetal bovine serum, 1% nonessential amino acids, and 1% streptomycin/penicillin. The cell lines were maintained at 37 °C in a humidified atmosphere of 10% CO_2_ and passed at 80% confluence.

### 2.3. Study of Intestinal Permeability by the Measurement of Transepithelial Electrical Resistance (TEER)

The bacterial effect on different epithelial barriers was evaluated by measuring TEER through Caco-2 and T84 cells using a REMS Autosampler (World Precision Instrument, Sarasota, FL, USA). Each cell line was seeded onto cell culture inserts (Transwell, 0.4-µm pore size, 6.5-mm diameter, polycarbonate, (Corning, New York, NK, USA) at 1.45 × 10^5^ cells/membrane. The membrane was maintained with a volume of 200 μL in the apical compartment and 800 μL in the basal compartment. The cell media were changed every two days. For this experiment, cells were grown in their respective complemented growth mediums and maintained in a humidified atmosphere (as described in Section 2.2.), until they reached their highest polarity. Two days before the experiment, the medium of the cells was changed to their respective mediums without antibiotic supplementation. TEER values were measured before the addition of bacteria (*t =* 0 h) and after 24 h of incubation. Bacterial suspension was added at a ratio of bacteria per cell of 40:1. The respective media were used as a negative control. The cells were challenged 3 h after the beginning of the coincubation with proinflammatory cytokines. Caco-2 cells were challenged with 100 ng/mL of tumor necrosis factor-α (TNF-α) (Peprotech, London, UK) on the basal side of the cells, and T84 cells were challenged with 50 ng/mL of interferon-γ (IFN-γ) (Peprotech, London, UK) on the basal side of the cells, and 1 ng/mL TNF-α was added on both sides. The TEER ratio was calculated as follows, and the bacterial effect of the strain was investigated by comparison with the controls.

$$TEER\;ratio=(\frac{TEER\;treatment\;t24}{TEER\;treatment\;t0}/\frac{TEER\;control\;t24}{TEER\;control\;t0})$$

### 2.4. Capacity of the Strains to Adhere to Intestinal Epithelial Cells

Quantitative analysis of bacterial adherence to differentiated Caco-2 cells and mucus-producing HT-29 MTX cells was performed. Each cell line was seeded into cell culture wells at 5 × 10^5^ cells/well. For this experiment, the cells were grown in their respective complemented growth mediums and maintained in a humidified atmosphere (as described in Section 2.2) until 21 days of the culture. Bacterial strains were grown overnight in their respective growth conditions (as described in Section 2.1), added to each well at a concentration of approximately 10^8^ CFU/well, and the plates were incubated at 37 °C in 10% CO_2_ for 1 h. After 1 h of incubation, adherent bacteria were detached from the cells using 0.5% Triton X-100 (Merck, Kenilworth, IL, USA). Each inoculum was serial diluted with de Man, Rogosa and Sharpe (MRS) medium and enumerated on MRS agar plates after 48 h of incubation at 37 °C.

### 2.5. Biofilm Structural Dynamics

Biofilm structural dynamics by the selected strains were observed, according to the protocol initially described by Bridier et al. [15] and using a LEICA SP8 high-content screening confocal laser scanning microscope (HCS-CLSM) at the INRAE MIMA2 microscopic platform [16]. For the kinetics, overnight bacterial cultures were diluted in order to obtain a concentration of 1 × 10^6^ CFU/mL and 200 µL of the resulting bacterial solution deposited into micro-clear microscopic-grade 96-well microplates (Greiner, Kremsmünster, Germany). After 1 h of incubation at 37 °C, bacteria were washed with MRS medium to keep only the adherent bacteria. Bacterial cultures were incubated for 0, 4 and 24 h at 37 °C. At each timepoint, bacteria were washed with saline. They were labeled with solutions of 1.66 µM SYTO9, a cell-permanent green dye that label all bacteria, and 10-µM propidium iodide (PI), a cell-impermeant nucleic acid dye that contrasts dead bacteria in red (Invitrogen, Carlsbad, CA, USA). SYTO9 and PI were excited with an argon laser set at 488 nm, and the emitted fluorescence was collected in the range 500 to 550 nm for the green signal and 650 to 70 nm for the red signal. For each biofilm analyzed, a series of 512 × 512 pixel images was acquired every micrometer on the whole biofilm thickness. Three-dimensional projections of the biofilm were reconstituted using IMARIS software. Quantitative biovolumes of the biofilms were extracted from HCS-CLSM images using ImageJ [17].

### 2.6. Comparison of Resistance to Stressful Conditions Depending on the Mode of Growth of the Lactobacillus Strains

#### 2.6.1. Planktonic Form

For this experiment, selected bacterial strains were grown overnight in their respective growth conditions (as described in Bacterial Strains and Growth Conditions). One milliliter of 1 × 10^6^ CFU of each bacterial culture was centrifuged, and the pellet was resuspended in 1 mL culture medium (control) or 1-mL culture medium at pH 2 (VWR) or 1 mL culture media containing 1% bile salts (Sigma Aldrich, Saint Louis, MO, USA) or 10-mM H_2_O_2_ (Sigma Aldrich, Saint Louis, MO USA). The cultures were incubated for 1 h at 37 °C. After a 48-h incubation (37 °C), bacterial cultures were enumerated on MRS agar plates by successive dilutions. After enumeration, the log reduction was calculated and compared to the control group (MRS).

#### 2.6.2. Biofilm Form

After overnight incubation, bacteria were centrifuged and diluted to a concentration of 1 × 10^6^ CFU/mL. Bacteria (200 µL) were deposited in 96-well plates (Corning, New York, NY, USA) and incubated for 1 h at 37 °C (adhesion step). The culture was washed with MRS medium to keep only the adherent bacteria and further incubated with fresh MRS medium at 37 °C for 24 h. After biofilm formation, the culture supernatants were removed and replaced with the medium (control) or medium at pH 2 (VWR) or media containing 1% bile salts (Sigma Aldrich, Saint Louis, MO, USA) or 10-mM H_2_O_2_ (Sigma Aldrich, Saint Louis, MO, USA)_._ The cultures were incubated for 1 h at 37 °C. After incubation, biofilms were resuspended in MRS medium by gentle pipetting and enumerated on MRS agar plates by successive dilutions. The log reductions were calculated and compared to those of the planktonic form.

### 2.7. Bile Salt Hydrolase Assays

The six *Lactobacillus* strains were tested for taurodeoxycholic acid (TDCA) and glycodeoxycholic acid (GDCA) hydrolase activities on MRS agar plates supplemented with either 0.5% TDCA (Cayman Chemical Company, Ann Arbor, MI, USA) or 0.5% GDCA (Merck Millipore, Burlington, CO, USA), following the protocol described by Moser and Savage [18]. Strains were grown for 16 h at 37 °C in MRS broth, streaked on MRS agar plates supplemented with 0.5% TCDA or 0.5% GDCA, and incubated at 37 °C for 48 to 72 h. Bile salt hydrolase (BSH) activity was detected by the observation of an iridescent halo below the precipitation of deoxycholic acid.

### 2.8. Analysis of Gamma-Aminobutyric Acid (GABA) in Culture Supernatants

Supernatant samples (500 µL) were extracted twice with 500 µL MeOH/H_2_O (50:50 v/v) in a sonicator bath for 10 min and then centrifuged for 10 min. Derivatization of the amino acid, GABA, was performed as previously described by Salazar et al. [19] and Collado-Gonzalez et al. [20]. Briefly, 350 µL borate derivatization buffer, 50-µL amino acid standard (or 20 µL supernatant samples), and 100 µL reconstituted ACQ (10-mM ACQ dry powder in acetonitrile) were combined. This solution was vortexed and then heated in a heating block for 10 min at 55 °C. Then, the samples were injected into an ultra-high performance liquid chromatography coupled to triple quadrupole mass spectrometry (UHPLC-QqQ-MS/MS) device. The chromatographic separation was achieved using a ACCQ-TAG^TM^ ULTRA C18 (2.1 × 100 mm, 1.7 µm) column [21,22] through a chromatographic gradient developed by applying a varying mixture of solvents A and B. Solvent A consisted of 50 mL of an aqueous solution (acetonitrile, formic acid, and 5 mM ammonium acetate in water) (10:6:84 *v*/*v*/*v*) diluted with 950 mL Milli-q water and the mobile phase; solvent B consisted of a mixture of acetonitrile and formic acids (99.9:0.1 *v*/*v*). The injection volume was 20 µL, and the elution was performed at a flow rate of 0.5 mL/min [21]. The MS analysis was performed in the multiple reaction monitoring mode (MRM) using the positive ionization mode. GABA was quantified based on a freshly prepared calibration curve (0.5–0.0039 µM) of the corresponding authentic standard (Sigma Aldrich, Saint Louis, MO, USA). The GABA concentration in the culture supernatant is expressed as nanogram per mL (ng/mL) [23].

### 2.9. Statistics

Results are expressed as the mean ± the standard error of the mean (SEM) of three experiments with duplicate determinations. The statistical analyses were performed using GraphPad Prism version 8 (GraphPad Software, San Diego, CA, USA). One-way ANOVA was carried out, followed by Dunnet’s or Tukey’s multiple comparisons test. For the three-factor analysis, a two-way ANOVA followed by Sidak’s multiple comparison test was used. *p*-values < 0.05 were considered significant.

## 3. Results

### 3.1. Modulation of Intestinal Permeability in Cellular Models

**Figure 1:**
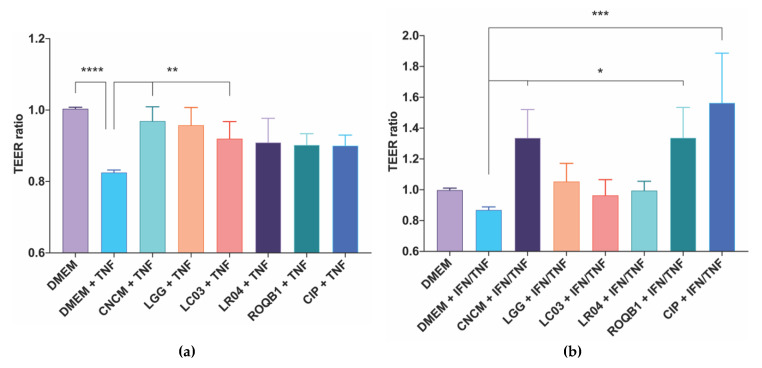
Screening of *Lactobacillus* species for their protective effects on intestinal epithelial cells permeability. TEER was assayed using (**a**) Caco-2 and (**b**) T84 cells grown on 24-well Transwell plates. TEER was measured before adding 6.10^6^ CFU of bacterial onto the apical surface for 3 h prior to treatment of the cells with a solution of TNF-α for Caco-2 cells and a mix of IFN-γ and TNF-α for T84 cells for 21 h at 37 °C and 10% CO_2_. TEER was measured a second time at the end of the 24 h of coincubation. Statistical analysis consisted of one-way ANOVA followed by Dunnet’s multiple comparison test. * *p* < 0.0332, ** *p* < 0.0021, ***p < 0.0002, and **** *p* < 0.0001.

We initially screened a panel of 50 strains for their impact on the transepithelial electrical resistance (TEER) of Caco-2 and T84 intestinal epithelial cells (Figure S1) based on evidence reported in the literature. The six *Lactobacillus* investigated in this study (Table 1) displayed the greatest effect on in vitro permeability, and the well-documented LGG was used as a positive control. The selected strains did not display the same level of beneficial effect on epithelial alterations induced by either TNF-α or IFN-γ in the two cell types (Figure 1). LC03 and CNCM had a strongly beneficial effect on Caco-2 permeability, similar to that observed with the positive control LGG (Figure 1a). The effect observed between the control and the strains on the T84 cell lines was less-marked, as the cells did not react to the same level to the mix of IFN-γ and TNF-α as did the Caco-2 cells with TNF-α. However, there was a clearly different response with the LGG and LC03 strains, and CIP and ROQB1 showed more promising effects than in the Caco-2 model (Figure 1b).

### 3.2. Capacity of the Strains to Adhere to Intestinal Epithelial Cells

**Figure 2:**
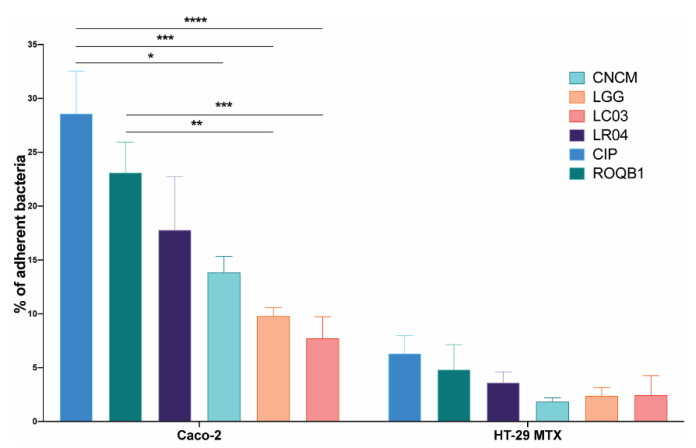
Adhesion of the six *Lactobacillus* strains on intestinal epithelial cells Caco-2 and HT-29 MTX. A bacterial suspension of a concentration of 10^8^ CFU/mL was applied to the intestinal cells. After 1 h of incubation, cells were washed to remove nonadherent bacteria and detached using 0.5% Triton X-100. Harvested solutions were then diluted in PBS X-1 and the bacteria enumerated. Results are expressed as the percentage of bacteria adhering to the intestinal cells. Statistical analysis consisted of two-way ANOVA followed by Sidak’s multiple comparison test. **** *p* < 0.0001, *** *p* < 0.0002, ** *p* < 0.0021, and * *p* < 0.0332.

We next characterized the capacity of our collection of *Lactobacillus* strains to adhere to intestinal mucosa by investigating their capacity to adhere to Caco-2 and mucus-secreting HT-29 MTX cells (originally isolated from a colorectal adenocarcinoma of human intestinal mucosa). The adhesive capacity of the strains varied considerably between the two cell lines, with a percentage of the adhesion of all strains up to five times higher to Caco-2 cells (Figure 2). There were also considerable differences between the strains, with CIP showing even higher levels of adhesion than the positive control LGG (*p* < 0.05).

### 3.3. Analysis of Biofilm Formation by the Selected Lactobacilli Using Confocal Laser Scanning Microscopy (CLSM)

**Figure 3:**
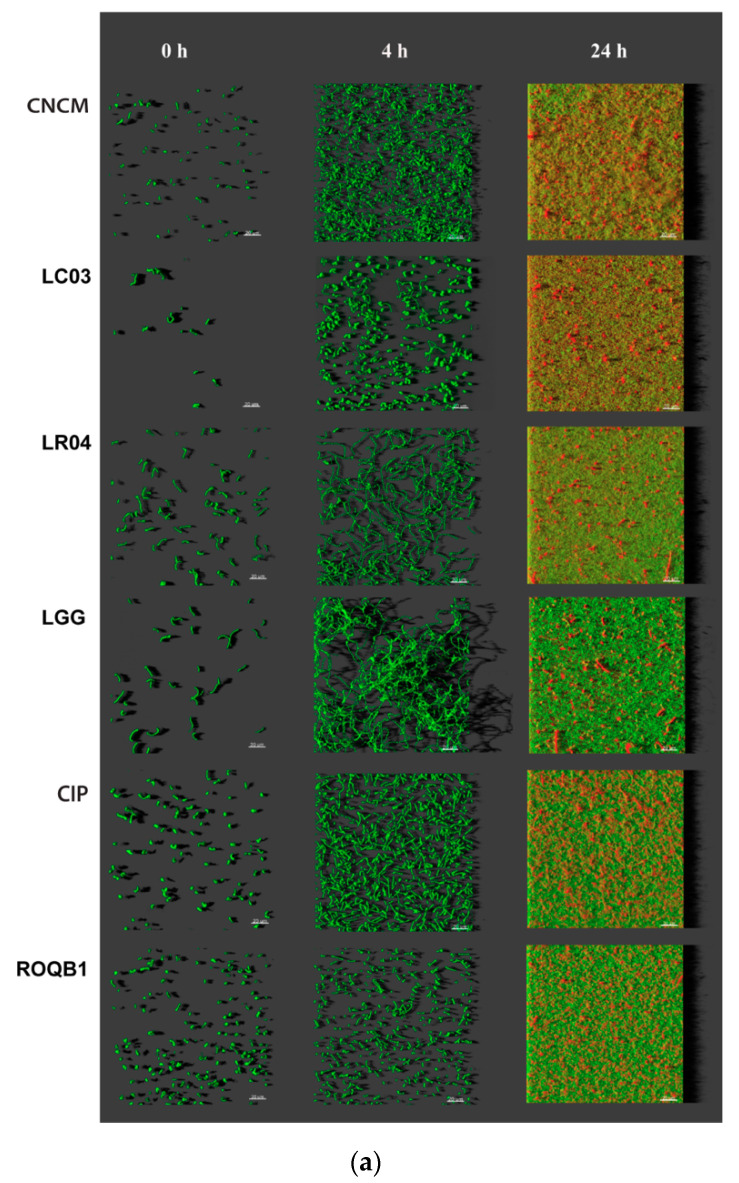

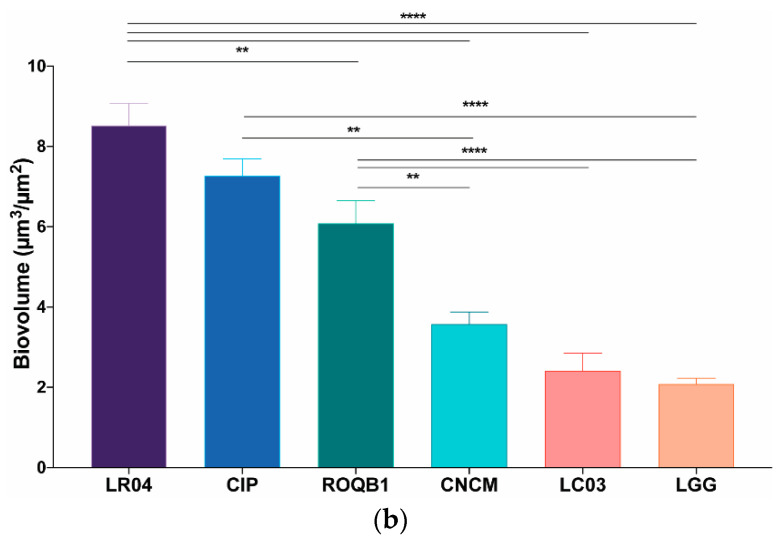
Observation of biofilm formation. (**a**) High-content screening confocal laser scanning microscope (HCS-CLSM) images at 0, 4, and 24 h after adhesion step. All bacteria are labeled in green (SYTO 9) and dead bacteria in red (PI). Images were treated using IMARIS software. The grey shadow to the right represents the thickness and relief of the biofilms. (**b**) Quantitative biovolumes of biofilms, at 24 h, were extracted by HCS-CLSM images using ImageJ. Statistical analysis consisted of one-way ANOVA followed by Tukey’s multiple comparison test. **** *p* < 0.0001 and ** *p* < 0.0021.

We analyzed the biofilm formation by the selected strains, in microscopic-grade 96-well plates, at 0, 4, and 24 h by using HCS-CLSM. The representative images were treated with IMARIS dedicated software (Figure 3a). Virtual shadow projections of the biofilm are represented on the right side of the images. The three isolates, LC03, LR04, and LGG, formed short chains that were visible after the adhesion (0 h). The chains formed by the LC03 strain were over-winded. For these three strains, the morphotypes observed were not specific to the adherent populations, as they were also observed for the planktonic bacteria in the stationary phase. After a short period of incubation (4 h), the adherent chains of the three isolates elongated to form an aerial structure. The LGG isolate showed a particular phenotype after 4 h with 3D aerial organization, which is likely associated with chain elongations and cohesion factors. The existence of sparse cells and aggregates labeled with PI indicate the presence of dead or damaged bacteria in those 24-h communities. The six *Lactobacillus* strains were able to form biofilms with different biovolumes of between 2 and 9 µm^3^/µm^2^ after 24 h of biofilm formation (Figure 3b, *p* < 0.05).

### 3.4. Comparison of Tolerance to Stressful Conditions Depending on Lifestyle

**Figure 4:**
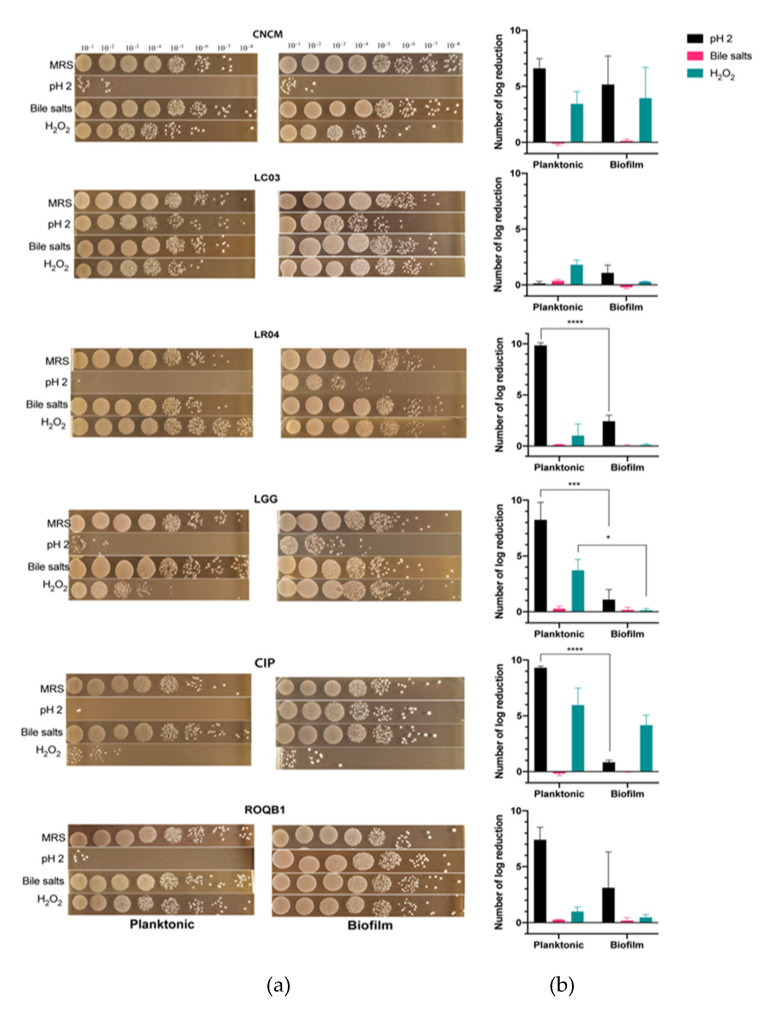
Survival test of selected bacteria to stressful conditions. Bacterial strains were grown under conditions to result in one of two lifestyles: planktonic or biofilm. The cells were then incubated in medium at pH 2 or in medium containing 1% bile salts or 10-mM H_2_O_2_ for 1 h. (**a**) Images represent the serial dilution plates that compare the different bacteria tested after the treatments with de Man, Rogosa and Sharpe (MRS) (control), pH 2, H_2_O_2_, and bile salts. (**b**) The log reduction after treatment was calculated by subtracting the bacterial concentration before incubation in the media at t = 0 h (MRS) to that at t = 24 h. Statistical analysis consisted of two-way ANOVA followed by Sidak’s multiple comparison test. **** *p* < 0.0001, *** *p* < 0.0002, and * *p* < 0.0332.

We compared the tolerance of the six *Lactobacillus* strains to stressful conditions as a function of their lifestyle (planktonic versus biofilm). From an overnight culture in the stationary phase, the bacterial strains were cultured for 1 h under hostile conditions using either oxidative stress (H_2_O_2_), low pH, or bile salts (Figure 4). Bacteria exhibiting either lifestyles were not statistically affected by the medium complemented with bile salts, suggesting their ability to tolerate high transitory concentrations of bile salts. In contrast, the growth rate of all strains was reduced at pH 2 (*p* < 0.05), with the exception of the LC03 strain. A protective effect of the biofilm lifestyle was especially marked at a low pH with LR04, LGG, and CIP strains for which there was a significant delta log (∆log), ranging from 8 to 5 (*p* < 0.05). Of note, the average of the ∆log for the ROQB1 strain was similar to those previously observed; however, the results were not significant because of the large variations that were observed between the replicates. Despite the fact that our isolates appeared less-affected by the oxidative medium than the acidic, we observed a benefit of the biofilm lifestyle for our control strain LGG with the H_2_O_2_ medium. Finally, these results also highlight that the LC03 strain possesses the highest tolerance to stressful conditions, either in the planktonic or biofilm lifestyles.

### 3.5. Bile Salt Hydrolase Assays

**Figure 5:**
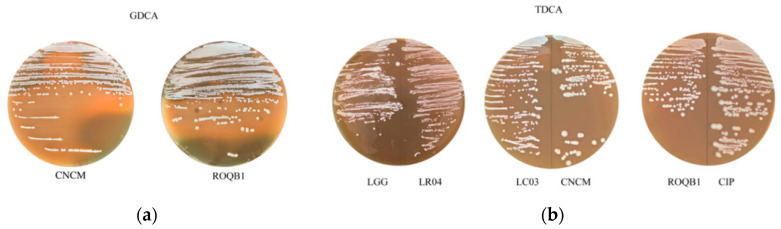
Bile salt hydrolase (BSH) activities. The six strains were tested for their (**a**) glycodeoxycholic acid (GDCA) and (**b**) taurodeoxycholic acid (TDCA) hydrolase activities. BSH activity was detected by the observation of an iridescent halo demonstrating the precipitation of deoxycholic acid. The presence or absence of the activity and growth capacity for each strain are summarized in Table 2.

We directly assessed the ability of the six selected strains to produce BSH in a colorimetric test on agar plates supplemented with either TDCA or GDCA. A large iridescent halo was observed with both the ROQB1 and CNCM strains (Figure 5), suggesting that they have the capacity to hydrolyze glyco-conjugated salts. None of the other four strains grew on 0.5% GDCA agar medium. Moreover, none of the strains showed TDCA activity, but, in contrast to the GDCA assay, they were all able to grow on 0.5% TDCA agar medium (Table 2).

### 3.6. Secretion of the Neurotransmitter γ-Aminobutyric Acid (GABA)

**Figure 6:**
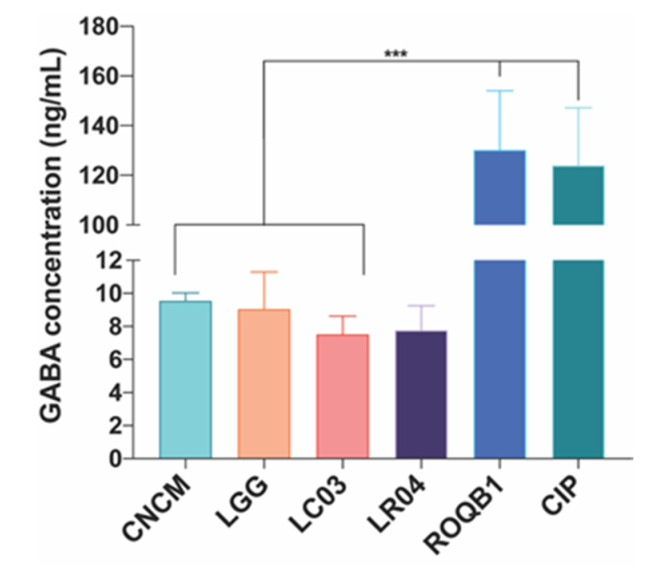
Production of γ-aminobutyric acid (GABA) after 16 h of incubation with separately inoculated *Lactobacillus* strains. Analysis was performed by UHPLC-QqQ-MS/MS, and the chromatographic separation was achieved by using an ACCQ-TAG^TM^ ULTRA C18 column. GABA was quantified based on the calibration curve (0.5–0.0039 µM). Results are expressed as nanogram per mL (ng/mL). Statistical analysis consisted of one-way ANOVA followed by Tukey’s multiple comparison test. *** *p* < 0.0002.

We tested the six *Lactobacillus* strains for their ability to secrete the neurotransmitter GABA. Culture supernatants were collected after overnight growth, and GABA was quantified by UHPLC-QqQ-MS. All strains were able to produce GABA, ranging from 7 to 130 ng/mL (Figure 6). Strikingly, ROQB1 and CIP exhibited the highest level, reaching 130.2 ± 23.86-ng/mL and 123.9 ± 23.29-ng/mL GABA.

## 4. Discussion

It is now well-accepted that alterations of the intestinal barrier are associated with chronic intestinal disorders, such as IBS; IBD [24]; and other conditions, such as obesity [25], liver diseases [26], and behavioral disorders [27]. Interactions between the various layers that constitute the intestinal barrier preserve normal permeability. However, several factors can disturb normal permeability, enabling the passage of toxins or pathogens from the lumen to the immune layer, leading to low-grade inflammation [28].

Various strategies have been explored for the treatment of these conditions, such as the use of probiotics. Indeed, numerous studies have demonstrated the potential benefit of *Lactobacillus* species for several conditions [29]. However, the positive effects of probiotics are often strain- and disease-dependent; thus, it is important to identify the strains that display the best and strongest beneficial effects [30].

Here, we evaluated six *Lactobacillus* strains for their ability to protect the epithelial layer from increased permeability (induced by proinflammatory cytokines) using TEER assays (Figure 1). We tested the potential probiotic effects of these strains on two types of cell lines: Caco-2 and T84 cells, to reflect different specific areas of the intestines. Caco-2 cells have been used for decades as an intestinal cellular model, as they have the ability to spontaneously differentiate, expressing morphological and biochemical characteristics of small intestine enterocytes [31]. In contrast, although T84 cells (less used in permeability assays) can also spontaneously differentiate, they differ from Caco-2 cells in that they express native intestinal characteristics, such as those found in undifferentiated crypt cells [32]. Our results confirmed the beneficial effects of the six *Lactobacillus* strains on Caco-2, with a significant effect of the CNCM and LC03 strains. In contrast, the CIP and ROQB1 strains showed the highest beneficial effect on T84 cells, whereas LC03, LR04, and the positive control LGG showed only slight effects. These results suggest that the enhancement of the permeability provided by the strains depends on the cellular localization.

The ability of bacteria to adhere to intestinal epithelial cells is a common and well-known criterion for probiotic selection. It has been proposed that the ability to adhere confers longer residence inside the gut, increasing the transit time, thus allowing them to exert their beneficial effects and interact with the host [33]. Of note, we recently described the role of a gene involved in the adhesive properties of lactobacilli (through pili) and the effects conferred to the intestinal barrier by the *L. rhamnosus* CNCM I-3690 strain [34]. The most common intestinal cell lines used for the determination of the adhesive capacity of bacteria are Caco-2 and mucus-producing HT-29 MTX [35]. The panel of the six tested strains displayed the ability to adhere to both cell lines (Figure 2). However, we observed a significant difference in the ability of the bacteria to adhere to the two cell lines, suggesting that mucus production may block adhesion-binding sites. Moreover, it is possible that the major determinants of bacterial adhesion, for the tested strains, are more highly associated with components of mature enterocytes than interactions with mucus glycoproteins [36].

Numerous studies have reported the beneficial properties of biofilm formation by *Lactobacillus* species by promoting longer survival of the bacteria on the host intestinal epithelium [37]. Moreover, given this advantage, recent studies have focused on biofilm-encapsulated bacteria based on two synthetic coatings, a new concept called “fourth-generation probiotics”. Bacteria exhibiting this particular status are better protected from the hostile environment and better at targeting specific localizations of the gut [38]. In this context, we tested the six *Lactobacillus* strains for their capacity to form biofilms on microtiter plates and to resist oxidative stress, low pH, and bile salts. All were able to form biofilms but of varying final biovolumes at 24 h, and they showed differing morphological changes during their growths (Figure 3). We compared the biofilm cells to their planktonic counterparts and found that the biofilm lifestyle clearly protected three of the tested bacteria (LR04, LGG, and CIP) better from low pH, confirming the advantage of this mode of growth in an acidic environment, such as that encountered in the gastrointestinal tract (GIT) (Figure 4). The formation of biofilms by microorganisms is strongly dependent on the bacterial species/strains; culture mediums; and conditions (temperature, surface properties, and pH) of the intestinal tract environment [3,39]. Moreover, the biofilm matrix represents 90% of the total biofilm and is responsible for its internal cohesion [40]. The matrix is composed of extracellular polymeric substances (EPS), of which some of their functions have been confirmed to be advantageous for the biofilm lifestyle and the enhancement of probiotic properties [41]. Moreover, EPS can be an important factor for the survival of bacteria in the microenvironment of the GIT. Indeed, the EPS content proportion has been correlated to bacterial tolerance to acidic environments and bile salts [42,43]. It is therefore important to better characterize biofilms under specific physiological conditions to achieve the optimal release and effects.

Primary bile acids are synthetized from cholesterol in the liver and become conjugated bile salts after their assembly with taurine or glycine amino acids. BSH enzymes, components of various species of the gut microbiota, hydrolyze and deconjugate glycine or taurine and generate secondary bile salts, which are more easily reabsorbed by the host [44]. BSH activity is now considered to be a suitable property of probiotics, as it may promote a tolerance to bile and survival of the bacteria in a hostile environment (such as that found in the intestinal tract) [45]. In addition, the beneficial impacts of certain BSH-producing bacteria have been reported for dietary lipid absorption, cholesterol lowering, and energy harvest [46,47]. Here, we aimed to compare the BSH activity towards either TDCA or GDCA-conjugated bile salts (Figure 5 and Table 2). We observed strong BSH activity on GDCA agar medium for two *Lactobacillus* strains, ROQB1 and CNCM, indicating their specificity for glyco-conjugated bile salts under these conditions. Of note, some of the strains were unable to grow in the presence of glyco-conjugated salt in the medium, demonstrating the low resistance of the strains to GDCA. It is worthwhile comparing these results with those obtained with the survival test (discussed above), as most of the strains were tolerant to higher concentrations of primary and secondary bile salts for a short period (Figure 3). These results suggest that the presence of glyco-conjugated bile acids, specifically, might be more toxic for the four *Lactobacillus* strains that lack BSH activity, with respect to long-term growth (48 h).

Probiotic strains are able to produce molecules that can influence the functioning of the central nervous system (CNS), behavior, and nociception [48]. GABA is a major neurotransmitter of the CNS, inhibiting the action potentials of neurons. Pérez-Berezo et al. [49] has shown that the inhibition of neuron activation by GABA is mediated by the formation of a lipopeptide, allowing diffusion of the neurotransmitter through the intestinal barrier. Furthermore, Bravo et al. highlighted the role of bacteria in gut-brain axis interactions and demonstrated a direct impact of *L. rhamnosus (JB-1)* on the GABAergic system through the vagus nerve [50]. Various studies focused on GABA-enriched functional foods with, for most, the use of an optimized-growth medium [51,52]. In this study, we confirmed that the selected *Lactobacillus* strains were able to produce GABA, but we were not able to state on the production of low or high amounts. However, normal concentrations of GABA in body fluids are found to be approximately 10 ng/mL in human plasma and 100 ng/mL in cerebrospinal fluid [53,54]. In addition, various studies reported a decrease of the GABA concentration in a variety of psychiatric and neurological disorders [55,56,57]. The imbalance between GABA and its glutamate precursor, an excitatory neurotransmitter, seems to be strongly associated to these conditions [58]. Overall, our findings provide new insights for the use of the *Lactobacillus* strains for the regulation of the GABAergic system and abdominal pain, commonly associated with intestinal barrier dysfunctions.

## 5. Conclusions

In conclusion, we demonstrated a significant protective effect of CNCM on both in vitro models of intestinal permeability. The LC03 and LR04 strains were better able to protect mature enterocytes of the small intestine, whereas ROQB1 and CIP showed a strong protective effect on crypt-like cells. We also compared the biofilm and planktonic forms of the bacteria and provided evidence of the advantages of the biofilm lifestyle in a low pH environment. Our data and others suggest that biofilm phenotypic assays should be systematically integrated in probiotic screening campaigns and provide new strategies for probiotic administration. Moreover, we found that the *Lactobacillus* strains were able to produce beneficial host/strain metabolites, and our findings on GABA production open new perspectives of characterization for their use in the management of gut-brain interactions. This step-by-step characterization provided interesting information of the *Lactobacillus* profile and highlighted the most promising strains for further evaluation in animal models of intestinal hyperpermeability.

## Figures and Tables

**Table 1:** Species, strain identifier, origin, and growth conditions.

*Lactobacillus* Species	Strains	Abbreviation	Origin	Growth Conditions
*L. rhamnosus*	LGG	LGG	Lesaffre	Facultative anerobia MRS 37 °C
*L. casei*	LC03 (DSM27537)	LC03	Probiotical	
*L. rhamnosus*	LR04 (DSM16605)	LR04	Probiotical	
*L. plantarum*	CNCM I-4459 (LP-Onlly)	CNCM	Novanat	
*L. plantarum*	CIP 104450	CIP	Institut Pasteur	
*L. paracasei*	ROQB1	ROQB1	Roquette Frères	

**Table 2:** Bile salts activities and tolerance. The absence or presence of bile salt hydrolase (BSH) activity and tolerance to 0.5% taurodeoxycholic acid (TDCA) or glycodeoxycholic acid (GDCA) on agar plates were evaluated.

*Lactobacillus* Species	Strains	TDCA Hydrolase Activity/Growth	GDCA Hydrolase Activity/Growth
*L. rhamnosus*	LGG	−/+	−/−
*L. rhamnosus*	LR04	−/+	−/−
*L. plantarum*	CNCM	−/+	+/+
*L. casei*	LC03	−/+	−/−
*L. plantarum*	CIP	−/+	−/−
*L. paracasei*	ROQB1	−/+	+/+

## Supplementary Materials

The following are available online at https://www.mdpi.com/2076-2607/8/7/1053/s1: Figure S1: Original screening of various bacterial species for their protective effect on intestinal cells’ permeability.
