# Supplementary material for: Evaluation of the Probiotic Properties and the Capacity to Form Biofilms of Various Lactobacillus Strains
Source: Microorganisms. 2020 Jul 15;8(7):1053. doi: 10.3390/microorganisms8071053 (PMC7409210; doi:10.3390/microorganisms8071053)
Supplement: Supplementary file 1 [file microorganisms-08-01053-s001.pdf]

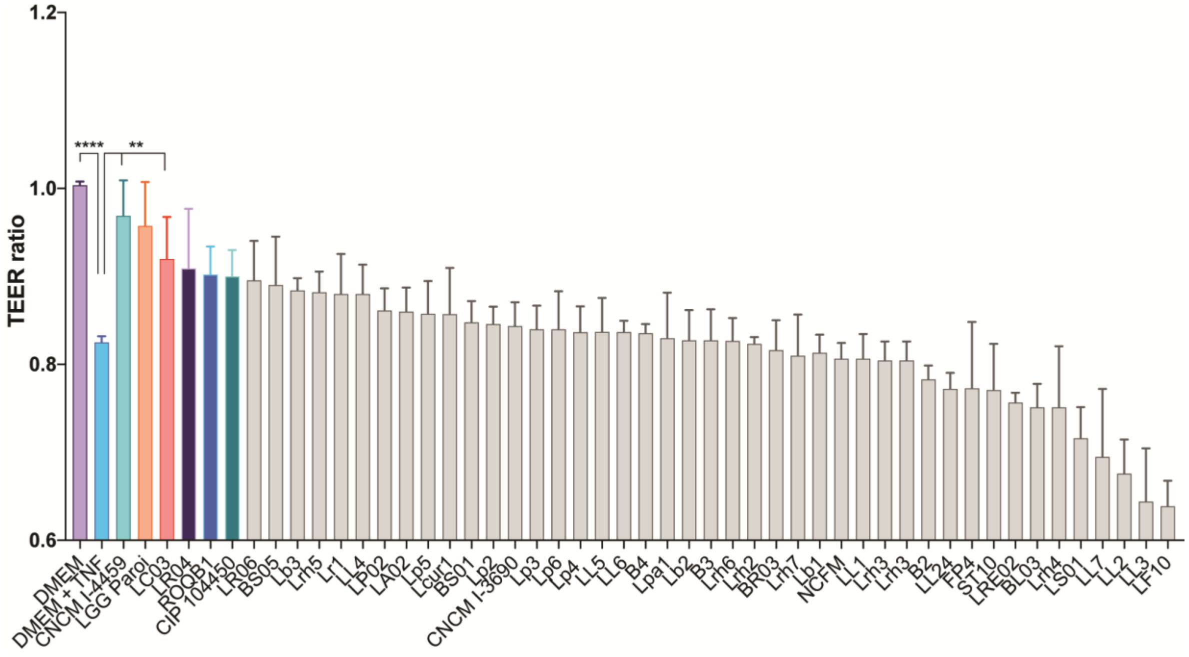

(**a**)

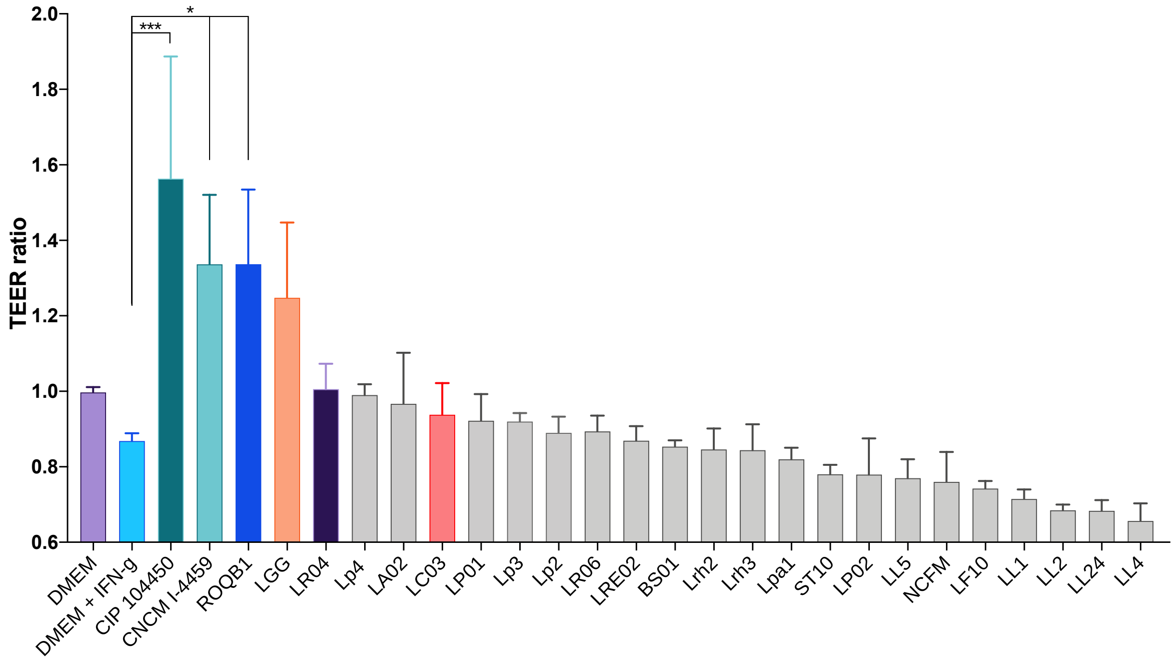

(**b**)

Figure S1. Original screening of various bacterial species for their protective effect on intestinal cells permeability**.** TEER method was used to evaluate the protective effect of the bacteria on the (**a**) Caco-2 and (**b**) T84 cell lines and provide an *in vitro* approach for the selection of potential probiotics. Statistical analysis consisted of one-way ANOVA followed by Dunnet's multiple comparison test. ****$P < 0.0001$, ***$P < 0.0002$, **$P < 0.0021$, *$P < 0.0332$.
